# A Light Wand to Untangle the Myocardial Cell Network

**DOI:** 10.3390/mps2020034

**Published:** 2019-05-03

**Authors:** Tania Zaglia, Anna Di Bona, Marco Mongillo

**Affiliations:** 1 Department of Cardiac, Thoracic, Vascular Sciences and Public Health, University of Padova, via Giustiniani 2, 35128 Padova, Italy; tania.zaglia@unipd.it (T.Z.); dibonaanna@gmail.com (A.D.B.); 2 Department of Biomedical Sciences, University of Padova, Via Ugo Bassi 58/B, 35122 Padova, Italy; 3 Veneto Institute of Molecular Medicine, Via Orus 2, 35129 Padova, Italy; 4 CNR Institute of Neuroscience, Viale G. Colombo 3, 35121 Padova, Italy

**Keywords:** heart, optogenetics, Channelrhodopsin-2, arrhythmias, heart network

## Abstract

The discovery of optogenetics has revolutionized research in neuroscience by providing the tools for noninvasive, cell-type selective modulation of membrane potential and cellular function in vitro and in vivo. Rhodopsin-based optogenetics has later been introduced in experimental cardiology studies and used as a tool to photoactivate cardiac contractions or to identify the sites, timing, and location most effective for defibrillating impulses to interrupt cardiac arrhythmias. The exploitation of cell-selectivity of optogenetics, and the generation of model organisms with myocardial cell type targeted expression of opsins has started to yield novel and sometimes unexpected notions on myocardial biology. This review summarizes the main results, the different uses, and the prospective developments of cardiac optogenetics.

## 1. Introduction of Optogenetics

If you were seeking information on “optogenetics” in a biology, physiology, or even neuroscience textbook published roughly ten years ago, you would be left wanting. Yet, a *pubmed* search using the same keyword in early 2019 returns more than 5000 hits, attesting to the tremendous success of this technique in biological and biomedical sciences. The recently coined word “optogenetics” summarizes the peculiar aspects of the methodology, which relies on the expression of microbial derived genes (-*genetics*) encoding one or more light-controlled ion channels or pumps (opsins) in the plasma membrane of a specific cell type. Although the word optogenetics now refers more comprehensively to both such *actuators* and any genetically encoded *sensor* emitting light, in response to variations of the intracellular environment (e.g., pH, voltage, Ca^2+^, cAMP to name a few), here, we will focus on optogenetics based on the use of channel-forming opsins. 

Most opsins are light-sensitive ion channel proteins which open rapidly upon illumination with visible light at a specific wavelength, causing redistribution of either cations (e.g., sodium, protons) or anions (e.g., chloride) across the membrane which, depending on the experimental conditions and cell types, results in cell depolarization or hyperpolarization [1,2]. The capacity of opsins to modify the cellular membrane potential has thus offered investigators unique tools to control, with minimal invasiveness on cellular homeostasis and cell-type precision, the quintessential property of excitable cells. 

The immediate success of optogenetics has prompted research of additional light-gated proteins present in nature, as well as the molecular modification of the ones already known, to expand the opsin toolkit with variants endowed with different spectral sensitivity (i.e., photoactivating light color), ion selectivity [3], activation/inactivation kinetics [4] or light-dependent conversion between on and off states [5]. As a result, more than 200 opsin variants are now available, as described in (for reference, see www.optogenetics.org) [1,6,7].

Optogenetics has primarily and most often been exploited in neuroscience to isolate (or interrogate) the function of specific neuronal types forming brain networks too densely juxtaposed to allow functional investigation with conventional methodologies. The method has revolutionized the study of brain connectivity in both normal and diseased conditions [8] and allowed association of the function of specific cell types to behavior [9,10,11,12], as well as the identification of pathogenetic mechanisms and potential therapeutic approaches in neuro-pathologies including, e.g., Parkinson’s disease [13,14,15] and retinal degeneration [16]. 

Unsurprisingly, “neuron+optogenetics” returns more than 3000 out of the total number of *pubmed* hits of optogenetics. While such abundant research has focused on the complex neuronal systems at the base of brain function, using various molecular tools, delivery strategies, and model systems [17], very little has comparatively been done to disentangle another intricated and equally vital cellular network, such as the heart. 

## 2. The Myocardium: A Complex Network of Excitable and Non-excitable Cells

**Figure 1:**
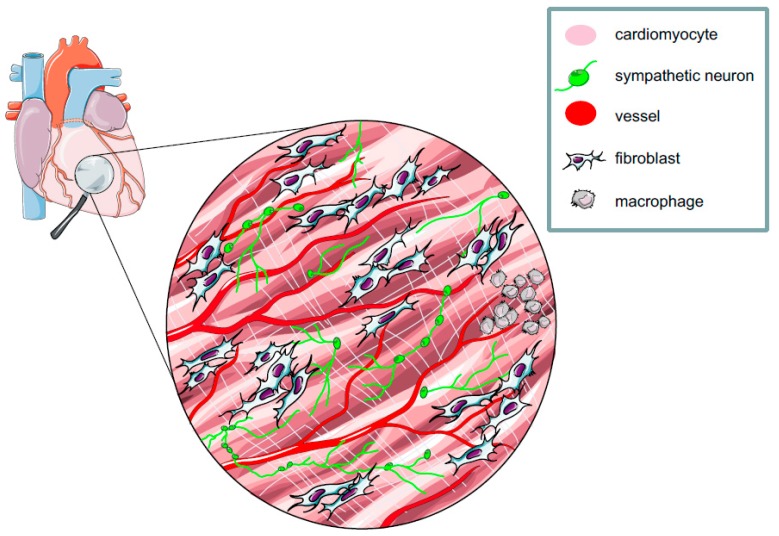
**The heart is a multicellular network of excitable and non-excitable cells.** Schematic representation of the myocardial network. As described in paragraphs 2 and 5, all cell types represented here have been targeted with opsins.

The heart is a strenuous worker contracting unceasingly through the entire life to provide oxygen and nutrients to all cells of the organism. The ability to sustain such crucial activity results from the cooperation of multiple specialized cell types, which are finely interconnected to one another in a well-defined arrangement [18,19]. The concept of the heart as a multicellular structure has been overlooked for a long time, as research focused on cardiomyocytes (CMs) (30–40% of all cardiac cells), responsible for both heart contraction (working CMs) and the conduction of electrical impulses (conducting CMs). This biased view caused the larger non-CM cell populations (60–70% of total cells in the heart), including excitable cells, such as neurons and vascular smooth muscle cells, and non-excitable cells (i.e., endothelial cells, fibroblasts, and immune cells) to sit in the background (Figure 1) [20,21,22,23]. However, these latter, seemingly silent cells have a key role in maintaining the electromechanical integrity of the heart and contribute to its adaptation to the intrinsic and extrinsic stimuli of daily activities, as well as in mediating myocardial remodeling upon tissue damage [24,25,26,27,28,29]. Interestingly, these functions are based upon various means of intercellular communication, including direct cell-to-cell interaction, secreted neurotransmitters or paracrine factors, and heterocellular electrical coupling, altogether orchestrating a well-regulated cross-talk between different CMs and non-CM cell types [30,31,32,33,34,35]. Thus far, such unique myocardial complexity has mostly been disarranged in *in vitro* studies on isolated cells, or analyzed upon pharmacologic or genetic perturbation of cell specific gene or protein functions [36,37,38,39,40,41,42,43], while strategies to interrogate specific cells in their native environment, the intact heart, have poorly been exploited. The view on the multicellular complexity of the myocardium is similar to the framework of the functional microcircuits of the brain, suggesting that optogenetics may represent, in molecular cardiology, as it does in neurobiology, an appropriate tool to disentangle heart circuitries. 

## 3. The Heart Goes to Optogenetics

After the initial run-in, while several groups provided evidence to support the use of Channelrhodopsin-2 (ChR2) as a tool to modulate the activity of mammalian neurons [8,44,45,46,47], optogenetics exploded in 2010 when it laureated Nature’s “*Method of the Year*” [48], and soon after touched the heart. The *charme* of this new technology enlightened molecular cardiologists, who first aimed at optically pacing the heart *in vivo*. On this trail, Bruegmann et al. [49] expressed ChR2 in mouse embryonic stem cell-derived CMs to test the method *in vitro* and generated transgenic mice with cardiac-specific expression of ChR2, which replicated, with light flashes instead of electrodes, epicardial pacing conventionally achieved in the electrophysiology (EP) lab. Simultaneously, ChR2 was expressed in the zebrafish heart and used to map pacemaker regions, while the yellow-light-activated chloride channel HaloRhodopsin from *N. pharaonis* (NpHR) was employed to inhibit cardiac function [50]. In addition, Jia et al. were able to control cardiac excitation and contraction with light by generating cx-43-coupled HEK cells stably expressing ChR2, using a so-called tandem-cell-unit (TCU) strategy [51], and demonstrated the feasibility of biological optical pacemakers. Taken altogether, these initial seminal works demonstrated that opsin targeting to the heart enables optical pacing of heartbeats in different regions of atria and ventricles, with minimal interference on the endogenous CM activity. These experiments were fundamental ‘proof-of concept’, which marked the beginning of the era of cardiac optogenetics, which was used later as a tool to delve into other aspects of cardiac physiology and pathology [52,53]. Cardiac optogenetic control required further research into the optical properties of the myocardium. By characterizing the attenuation function of ChR2-activating blue light across the heart wall, it was possible to photoactivate different tissue volumes. This was used to experimentally determine the liminal cell number required to generate extra-systolic foci, which directly assessed the threshold to overcome the protective effect of electrotonic coupling between CMs. Before optogenetics, such a physiological concept was only theoretical and inferred through numerical modeling. By applying the photoactivation assay to different regions of normal and ischemic hearts, optogenetics revealed the high arrhythmogenic potential of ectopies occurring during acute myocardial ischemia in the right ventricular outflow tract [52]. 

Preclinical research using cardiac optogenetics has soon aimed at developing therapeutic concepts, ranging from light-operated termination of arrhythmias to the development of biological pacemakers for cell therapy, to cardiac resynchronization [54]. In 2014, Entcheva and, simultaneously, Trayanova asked cautiously whether optogenetics would, in the following years, maintain the promise of restoring healthy heart rhythm in patients, and provided the groundwork for computational modelling of light-assisted modulation of normal and arrhythmic heartbeats [55,56]. In the following five years, modelling of cardiac optogenetics evolved to address opsin spectral sensitivity and illumination protocols, and was computed on realistic imaging data from patient’s hearts [56,57]. In parallel, the path towards clinical translation of optogenetics was signed by several reports testing suitable opsin delivery strategies [58], and showing in preclinical models the feasibility of optical antiarrhythmic therapy [53,59,60,61]. 

## 4. All-Optical: Optogenetics, Optical Mapping, and Optoelectronics

**Figure 2:**
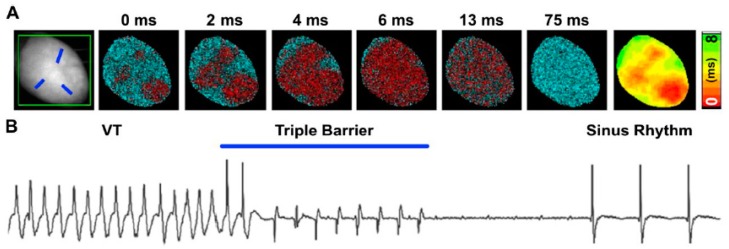
**Optogenetic defibrillation of mouse ventricular tachycardia via patterned illumination.** (**A**) Pattern of optogenetic stimulation designed with three discrete sites simultaneously illuminated (referred to as Triple-barrier illumination strategy), as indicated by the blue traits on the fluorescence image shown in the left panel. The remainder pseudocolored maps show propagation of light-induced excitation in ChR2-expressing transgenic mouse heart, obtained with the red-shifted voltage sensitive dye (Di-4-ANBDQPQ). (**B**) ECG signal demonstrating interruption of ventricular arrhythmia and restoration of the normal sinusal rhythm upon triple-barrier pattern optogenetic stimulation (modified with permission from Crocini et al.).

The possibilities offered by optogenetics in the understanding of physiologic and pathologic mechanisms of heart diseases have prompted the combination of light-activation with optical mapping of voltage or Ca^2+^ dynamics, on one hand, and methods to achieve spatially tunable light delivery on the other. All-optical investigation of cardiac electrical activity in ChR2-expressing hearts is possible by using voltage sensitive dyes, with excitation and emission spectra separated from the opsin activating light [52]. Combination of a temporally accurate actuator with high speed optical voltage mapping has allowed interruption of sustained arrhythmic waves by illuminating spatially defined areas based on the wave dynamics (Figure 2) [54,59,61]. In addition to voltage, given the central role of Ca^2+^ in cardiac function, optogenetics has been combined with Ca^2+^ sensitive dyes to monitor the second messenger dynamics upon photoactivation of specific CM subpopulations in isolated hearts [62]. Since alterations in Ca^2+^ dynamics are associated with arrhythmogenesis, all-optical depolarization/Ca^2+^ detection has been employed *in vitro* to develop a semiautomated antiarrhythmic drug screening platform [63,64,65,66]. 

The development of light crafting tools to define dynamically and at high resolution the localization of the incident photostimuli is rapidly endowing cardiac optogenetics with ways to both generate and interrupt arrhythmic waves [67,68]. The combination of techniques used in these studies inform on arrhythmia mechanisms and help refining the therapeutic electrophysiologic protocols routinely used in the clinic to interfere with arrhythmic circuits. In parallel, the evolution of light emitting device technologies has miniaturized light sources, allowing *in vivo* optogenetics in freely moving animals [60,69]. Further technologic improvement has allowed to couple arrhythmia-detecting telemetry ECG with the activation of implanted light sources and develop hybrid bioelectronic photo-defibrillating devices [70]. Finally, all-optical approaches have used opsins *in vitro* to implement automated platforms tailored for multiplexed, high-yield drug screening or cardiotoxicity studies [62,64]. These examples underline the bond between optogenetics and progress in optoelectronics, which will likely expand the applicability of the tool for cardiac research and therapy. 

## 5. Optogenetics Targets Specific Heart Cells

As discussed above, the distinctive advantage of optogenetics over conventional methods to control cellular activity is cell type specificity. To achieve this goal, either of two main strategies have commonly been used: (i) expression of opsins under control of a cell-specific genetic driver (e.g., cell type specific promoters such as α-MyHC) or (ii) cell-selective tropism of viral vectors incorporating the opsin transgene [54,71]. While each of the two strategies has its own advantages relative to the experimental needs, which include on one hand the generation of stably expressing mouse strains, and on the other the use of different model systems (including larger mammals, or human-derived cells), they increase the versatility of optogenetics. These strategies have allowed investigations of selected cell types forming the myocardial network, some of which are summarized below.

**Figure 3:**
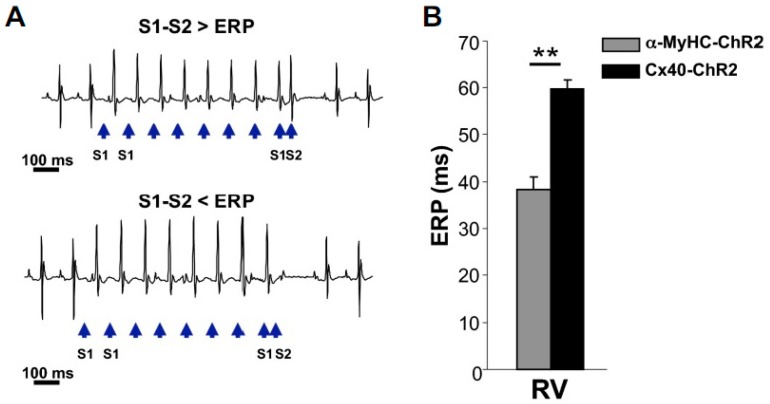
**Optogenetics allows selective interrogation of Purkinje fibers electrophysiology *in vivo*.** (**A**) Optical programmed stimulation using the extrastimulus (S1–S2) protocol to measure the PF effective refractory period (ERP). Blue arrows indicate the light pulses. (**B**) Comparison of the ERP of ventricular myocytes and PF as obtained with photostimulation of the RV surface in α-MyHC-ChR2 (gray bar) and Cx40-ChR2 (black bar) mice, respectively. Bars represent s.e.m. (**, *p* < 0.01; n = 10 α-MyHC-ChR2 mice and n = 5 Cx40-ChR2 mice) (modified with permission from Zaglia et al.).

*Conduction system cells*. With respect to the excitable cell component of the myocardium, the working and conducting CMs coexist, with the former making up most of the muscular mass of the heart, and the latter organized in a tree-like network which is anatomically defined at the septal root (His bundle) and infiltrates, with progressively narrower branches, the muscular matrix of the cardiac walls [72]. The distal sector of conduction system, the so-called Purkinje fiber (PF) network, has a fundamental role in physiological heart activation, and despite its dysfunction has been centrally implicated in arrhythmogenesis [73,74], its distribution in the heart sub-endocardium has limited specific investigation to destructive approaches (e.g., chemical ablation) [75]. PF-targeting of ChR2 was achieved by Zaglia et al. using the cx-40 promoter and allowed to optically pace the heart through distal PF activation [52]. The cell-specific approach was used to assess *in vivo* electrophysiological properties of the intact conduction system, i.e., refractoriness (Figure 3), a parameter hardly possible to determine with other methods. In addition, an estimate of the minimal number of cells needed to activate conducted beats from the PF network was obtained, proving in intact hearts the previously simulated result [76,77] that, due to their cable-like arrangement, reduced dispersion of depolarization increases the susceptibility of PFs to originate ventricular beats [52]. 

*Cardiac neurons*. Regarding cardiac excitable cells, the neuronal component of the myocardium has been neglected for a long time. However, heart function is continuously tuned by the balance between the activity of parasympathetic and sympathetic autonomic nervous inputs, which match heart rate and contractility to the instantaneous requirements of the organism. While acetylcholine, released by parasympathetic fibers, at the SinoAtrial (SAN) and AtrioVentricular (AVN) Nodes, reduces heart rate, noradrenaline (NE), discharged by the sympathetic neurons (SNs) innervating both the atria and the ventricles, is responsible for positive chronotropic and inotropic responses *via* β-adrenoceptors (β-AR) [78]. Alterations in the pattern of cardiac innervation and β-AR signaling have been linked to cardiac arrhythmias and, unsurprisingly, β-AR are targets of key cardiovascular therapeutics [79,80]. Despite this evidence, the mechanisms underlying autonomic control of heart function, including the biophysics of intercellular neurocardiac communication, have been somewhat poorly addressed. Recently, optogenetics started to light up the aspects of neurocardiology, and in 2015 Wengrowski et al. generated a novel mouse model expressing ChR2 under control of the SN promoter Tyrosine Hydroxylase (TH). Consistent with the effects of NE released by SNs, epicardial photostimulation of isolated hearts resulted in a positive chronotropic response, increased inotropism, and reduced Action Potential Duration (APD). In addition, fast pacing elicited sustained arrhythmic events [81]. By using the same murine model in an open-chest configuration, Prando et al. developed a optogenetic neurocardiac coupling assay which demonstrated *in vivo* that SNs communicate to target CMs in a ‘quasi-synaptic’ fashion, thanks to the establishment of a specific interaction site, in which NE is released in a restricted intercellular cleft [82]. More recently, the Kay group has targeted ChR2 to cholinergic neurons, contributing the specular concept that dense innervation of the SAN allows parasympathetic acetylcholine to directly control heart rhythm with a high temporal resolution [69]. That optogenetics can be used to study neuronal influence on heart physiology has also been demonstrated in different model systems, e.g., *Drosophila melanogaster*, upon expression of neuronally-targeted ChR2 in SNs, showing the photoactivated increase in heart rate [83]. 

The increasing attention to autonomic neuromodulation, as a strategy to target the arrhythmogenic effect of SNs, especially in conditions favoring electrophysiologic vulnerability, such as myocardial ischemia or heart failure [84,85], prompted the use of optogenetics to achieve optical SN inhibition. This concept was successfully proved by Yu et al. who delivered viral vectors transducing the inhibitory light-sensitive opsin, archeorhodopsin (ArchT), to the left stellate ganglia neurons of dogs. Optogenetic modulation could reversibly inhibit SN activity and was effective in preventing ventricular arrhythmias upon myocardial ischemia [86]. The use of autonomic neuron-targeted opsins, combined with implantable devices to achieve local illumination of cardiac sympathetic and parasympathetic neurons (e.g., [69]) is, in prospect, transferable to clinical applications as a flexible tool for heart neuromodulation. 

*Cardiac non-excitable cells.* A large part of the myocardium is constituted by non-CM cell populations, mostly interstitial cells or components of blood vessels, all of which fall into the general classification of non-excitable cell types, for the lack of activation mechanisms (e.g., voltage dependent ion channels) responding to the variation of membrane potential. As such, the contribution of these cells to myocardial electrophysiology has, for a long time, not been supposed. Recent research, mostly conducted *in vitro* or *in silico* [51,57,87,88], and less frequently using electrophysiological mapping of the intact heart [89] has, however, taken into account the electrophysiological role of the cell types which, although not native parts of the CM syncytium, may electrically couple to it. These instances include the electrotonic effect of fibroblasts intertwined to CMs in the intact myocardium, or, e.g., the conditional coupling of inflammatory cells, like macrophages (MΦ), to CMs. The use of opsins, by allowing cell specific modulation of the membrane potential, has enabled, as exemplified in more detail below, the uncovering of emerging properties of nonexcitable cells in the conduction of cardiac activation waves. 

*Cardiac fibroblasts.* Fibroblasts are the most abundant cardiac interstitial cells which are commonly recognized as having a structural role of generating the scaffold sustaining the complex myocardial architecture. While it is difficult to define the signature of cardiac fibroblasts (CFs) in terms of cell origin and surface markers [90,91], researchers agree that correct CF function is key for the maintenance of the healthy extracellular matrix, and in its remodeling after tissue damage [92]. In addition, CFs modulate the activity of CMs, both through the release of extracellular factors [23,34,35], and *via* direct mechanical or, interestingly, electrical interaction. This latter concept collides with the traditional notion of CFs as electrically inert "insulators" with regards to the cardiac activation wave. However, evidence is recently accruing which demonstrates that: (i) CFs express, although at low levels, cx-43, cx-45 and cx-40 [37,38], which are the CM connexin isoforms, and consistently mediate both ‘CF-CF’ homo- and ‘CF-CM’ hetero-cellular coupling; (ii) in certain sectors of the conduction system, i.e., the SAN [31], CFs are functionally connected to CMs; (iii) CFs may change their membrane potential during mechanical perturbation [93], and (iv) due to their low membrane capacitance and a high coupling resistance, CFs may function as slow conductors of electrical signals for long intramyocardial distances. 

Although they are “non-excitable cells”, the aforementioned properties may change upon damage inducing CFs activation, resulting in membrane hyperpolarization, increased outward current density and membrane resistance [94,95] and enhanced cx-43 expression [96,97]. Excitingly, activated CFs not only contribute to the structural, but also to the electrophysiological myocardial remodeling, synergizing to explain the increased arrhythmogenic vulnerability [98]. Altogether, this justifies the appeal of CF optogenetics as a tool to illuminate the obscure aspects of “CF–CM” communication. 

When using CF targeted optogenetics, the main problem research had to overcome was the lack of a fibroblast-specific marker. At the time, expression of opsins was achieved in isolated cell lines (e.g., HEK293, 3T3) by stable transfection or infection with specific adenoviral vectors, which allowed study of “CF–CM” coupling *in vitro*. As proof of principle, expression of ChR2 or ArchT was used to demonstrate that light-assisted modulation of CF membrane potential may affect the coupled CM electrical properties. Although the *in vitro* context is far from the complexity of the intact myocardial network, and stable cell lines may not faithfully replicate the peculiar properties of CF, in a recent report, hetero-cellular coupling between myocytes and non-myocytes has been assessed in the intact damaged myocardium using genetically encoded voltage sensitive fluorescent proteins (i.e., falling into “optogenetics” in its broader sense) [99]. The results of these studies have opened a novel view on interstitial myocardial cells, suggesting optogenetics may represent a tool to investigate it further.,The results of these studies open a novel view on interstitial myocardial cells. In a somewhat counterintuitive approach, the optical modulation of CF membrane potential may be exploited in future studies for antiarrhythmic purposes or resynchronization therapy, tasks currently reserved for the noble excitable components of the heart [71,87,100]. 

*Vascular cells.* Several cardiac pathologies are caused or accompanied by defects in the myocardial vascular system, which therefore represents a fundamental point of intervention for the therapy of heart diseases. The direct investigation of the cellular components of coronary arteries and capillaries in the intact heart *in vivo* has been hampered by their anatomy, which makes them difficult to reach, except for large epicardial vessels. Recently, Wu et al. generated a mouse model expressing ChR2 selectively in the excitable, contractile component of blood vessels, the vascular smooth muscle cells (SMCs), and demonstrated that spatially restricted photoactivation could be used to trigger localized vasoconstriction [101]. Given that the control over vasomotor tone is grounded on the coordinated function of both the contractile smooth muscle and the non-excitable endothelial cells (ECs), in analogy to the “CF–CM” hetero-cellular coupling in the working myocardium, the study of intercellular communication within the vessel wall *in vivo* is of paramount physiologic importance. Remarkably, ECs may modulate SMCs function both through the release of paracrine factors (either vasoconstrictors, i.e., ET-1 TxA2, or vasodilators, i.e., NO) and through direct electrical coupling mediated by different connexin isoforms [102]. Furthering the study of vascular physiology *in vivo*, Zhang et al. generated mice expressing ChR2 in ECs, and assessed the role of selectively modulating EC membrane potential on SMC contractile tone, demonstrating that photostimulation of ECs elicited in the intact excised heart “fast, robust, reproducible and long lasting vasoconstriction” [103]. Although currently vascular cell optogenetics has had very little use, it is foreseeable that the method will shortly yield important advancements in vascular cell physiology. 

*Cardiac inflammatory cells.* Inflammation plays a central role in heart pathology and the study of cardiac inflammatory cells represents an expanding branch of cardiovascular research. Although in a simplistic view, inflammatory cells are recruited from the bloodstream to the site of tissue damage, resident cell populations have commonly been identified in all organs, including the heart. Resident cardiac macrophages (MΦ) have a key role in both the early response and subsequent phase of tissue repair, e.g., following myocardial ischemia [104]. Detailed histopathological analysis of the myocardium identified MΦ interspersed within the ventricles and in proximity of the AVN and conduction system [105]. Such peculiar topology prompted investigation of whether MΦ could have, in addition to its best-known role in the inflammatory response, effects more directly related to the conduction of electrical impulses in the heart. That MΦ could electrically couple to CMs was initially predicted based on computational modelling, and subsequently demonstrated to occur through gap junctions, allowing synchronous depolarization of the coupled cells [106]. Interestingly, expression of ChR2 in MΦ allowed demonstration that modulation of MΦ membrane potential had, conversely, the effect of facilitating AV conduction. Consistently, conditional deletion of cx-43 in MΦ or the congenital absence of these cells delayed AV conduction [107]. Thus, optogenetics allowed identification of a previously unrecognized and unpredictable function of MΦ in the heart and indicated that these cells participate directly in normal and altered conduction of cardiac electrical signals.

## 6. Conclusions

Here, we have briefly covered the applications of optogenetics in the study of cardiac physiology and pathology. This summary is by no means comprehensive and we apologize to the many colleagues who contributed to the field but have not been cited. In our opinion, cardiac optogenetics is still at a rather naïve stage, but is rapidly progressing beyond being a stylish tool for heart pacing. The opsin toolkit includes a large number of variants with specific features, only a few of which have been used in cardiac optogenetics. For instance, light control of intracellular signaling may be exploited in the study of myocardial development and pathologic remodeling (e.g., hypertrophy, failure). An open field of study which will benefit from the use of optogenetics is the understanding of the roles of the many different cell populations forming the heart, explored in their intact environment. With the pace of current progress in molecular biology and light crafting technology, a multicolored heart with every cell type activatable with its appropriate light beam to interrogate the myocardial network in healthy and diseased hearts can be pictured in the near future. 

## Abbreviation

α-MyHC	alpha-myosin heavy chain
ArchT	archeorhodopsin
AVN	atrioventricular node
APD	action potential duration
β-AR	β-adrenoceptors
ChR2	Channelrhodopsin-2
CF	cardiac fibroblast
CM	cardiomyocyte
cx	connexin
EC	endothetial cell
ECG	electrocardiography
EP	electrophysiology
ERP	effective refractory period
ET-1	endothelin-1
MΦ	macrophage
NE	noradrenaline
NO	nitric oxide
NpHR	*N. pharaonis* HaloRhodopsin
PF	Purkinje fiber
RVSAN	right ventriclesino atrial node
SMC	smooth muscle cell
SNs	sympathetic neurons
TCU	tandem cell unit
TH	tyrosine hydroxylase
TxA2	thromboxane-A2.
